# Discourse analysis of health providers' experiences using service design

**DOI:** 10.1002/nop2.191

**Published:** 2018-08-21

**Authors:** Trude Fløystad Eines, Elin Angelo, Solfrid Vatne

**Affiliations:** ^1^ Molde University College Specialised University in Logistics Molde Norway; ^2^ Norwegian University of Science and Technology Trondheim Norway

**Keywords:** discourse analysis, focus group, innovation, nursing home, service design

## Abstract

**Background:**

Municipal healthcare organizations are facing increasing challenges to developing more cost‐effective organizations and services, particularly in nursing homes. The background for this article is an attempt in this concern; implementing service design in a nursing home.

**Aim:**

The aim of the study is to explore nurses and nurse assistants' experiences using service design in a nursing home.

**Design:**

A qualitative design was applied.

**Method:**

Data were collected through focus group interviews with 17 nurses and nurse assistants in 2015. Thematic discourse analysis was conducted to analyse their discussed experiences.

**Results:**

The employees had positive attitudes towards incorporating a service design approach that focused on the patients' needs, which thus encouraged creative solutions and promoted employee involvement. The study shows that involving service designers and employees in the entire process, from planning to implementation of new and innovative solutions, requires closer collaboration between the service designers, managers, and employees to achieve the final goal. We consider that the results of this study will be of relevance to the future development of health care and nursing in nursing homes.

## 1 INTRODUCTION

Municipal healthcare organizations are under unprecedented financial, social, and political pressures to develop more robust healthcare systems for better aligning current and future services (Roberts, Fisher, Trowbridge, & Bent, 2016; Thakur, Hsu, & Fontenot, 2012). Because of existing inefficiencies, the Ministry of Finance in Norway (2015) has stressed the importance of a more market‐oriented approach in developing public services. Previous research has indicated that future health services should be more people‐ and patient‐centred, with a focus on the patients' needs in the planning of health care (Roberts et al., 2016; Ministry of Health and Social Services in Norway, 2008, 2011, 2012).

Innovation in health care describes implementing changes that focus on the patient's needs by enabling health professionals to work smarter, faster, better, and more cost‐effectively (Thakur et al., 2012). Service design is a new discipline in the public health services that has been receiving increasing attention in recent years (Architecture and Design Academy in Oslo (AHO)/Norwegian Association (KS), 2015). Service designers prioritize obtaining a deep understanding of the users' needs and challenges to enable them to develop more comprehensive and effective solutions (Roberts et al., 2016; Stickdorn & Schneider, 2011). At the start of a project, service designers spend time talking with all the people affected by a service to search for new solutions to existing problems. The service designers then start prototyping different solutions and testing them with those involved in the services. Multiple ideas are put into action, often on a small scale, to learn something about the potential solution. After a series of critical evaluations, an optimized solution emerges that can be scaled for the implementation (Roberts et al., 2016; Stickdorn & Schneider, 2011). Service design processes thus need to be user‐oriented, co‐created, visually prepared and continuously tested (Stickdorn & Schneider, 2011). Implementing service design in the development process of health services means applying a framework that could be essential for achieving innovation in different healthcare systems (Ferreira, Song, Gomes, Garcia, & Ferreira, 2015).

Previous research has focused on employees' participation and experiences with service design in various ways. For example, Lee (2011) found that healthcare systems have recognized the importance of how patients and relatives experience the services provided and have thus drawn knowledge from the service provision of other services, such as restaurants and hotels, to treat patients as guests (Lee, 2011). Lee (2011) and Morrison and Dearden (2013) found that healthcare providers needed to focus on understanding the patients' knowledge and evaluating the healthcare services to create better patient‐centred health care. User participation has expanded beyond surveying people to gather feedback about services to include meaningful partnerships facilitated through co‐learning, active collaboration and shared power and decision‐making in healthcare (Palmer et al., 2015). If both care providers and patients experience meaningful participation, they will aim to improve health services in a way that reflects both groups' priorities (Morrison & Dearden, 2013). Using service design in American hospitals led to an improved knowledge base among physicians, nurses, and staff members (Ding, 2015). Involving both patients and staff members in a service design process was also found to reduce the readmission rates for hospitals in Florida (Ding, 2015). Service designers could also help healthcare providers to elicit use of different levels of service segments, which is much needed in complex healthcare systems (Clatworthy, 2012; Dixon & Verma, 2013). Additionally, Palmer et al. (2015) found that implementing new interventions that were developed with the involvement of healthcare providers and patients with mental illnesses could be staggered by the management of the institutions to manage practicalities and logistics. The aim of the study is to explore nurses and nurse assistants' experiences using service design in a nursing home.

### 1.1 The project—Organizing nursing homes for the future

The nursing home called “Knausen,” experienced increasing demands for improved service quality and economic balance and for the recruitment of skilled healthcare professionals. As a result, in 2014, the management employed a private service design firm to build a smarter organization. Three service designers observed the nursing home services for 2 days and organized workshops with patients, relatives, employees, and various volunteer organizations to gain insights about their experiences and needs. The findings from this initial period of the project indicated that the patients felt that insufficient daily activities were provided, while the employees explained the difficulties they experienced prioritizing active care. Dialogue exchanged during the various workshops with the staff gave the service designers insight into the necessity for reorganizing the employees' roles, tasks, and responsibilities and generated ideas about how to build up a new model that used the employee's skills more effectively. This process resulted in a team‐based solution with clearly defined roles for the nurses and nurse assistants which included the following: (a) a team for general care to take care of the patients' daily needs, such as taking a shower, getting dressed, and receiving nutrition; (b) a team for nurses and nursing interventions involving leadership in medical care, medication, and nursing procedures; (c) a team for organizing well‐being and life quality activities, such as music, playing cards, training, and walking outside; and (d) a service team: for supporting all types of services, including cleaning, filling up equipment for the patients and staff, and organizing meals. The proposed method for working in these four teams was tested from May–September 2015.

## 2 THE STUDY

### 2.1 Design

This study used a qualitative design to examine the research participants' discussions. Qualitative inquiries seek understandings that cannot be counted or measured, but that needs to be interpreted and analysed in line with the context, content, persons, and consequences. With a discourse‐oriented approach, the study focused on the relationship between language and social reality by examining how the participants explained their experiences of the process. The verbal dialogue and construction of service design in health care between the professionals and between the professionals and the users, is crucial at all levels of a project, from more distant political levels to close practical levels, between the professionals, or the professionals and the users (Stickdorn & Schneider, 2011). Our research thus focused on the participants' language, concerning, for example, the choice of words, metaphors, and themes to explain the hypotheses, predictions, and meanings. Such a qualitative, text‐oriented focus is crucial when investigating the meanings of service design among employees in health care and examining the world of health care that is constructed through the language used like in this project (Wertz et al., 2011).

### 2.2 Method

Over the last 20 years, discourse analysis has gradually been accepted as an important method in healthcare research (Buus, 2005). Discourses authorize and encourage inquiry into the framing and understanding of prevailing structures of knowledge and power (Kunyk, Milner, & Overend, 2016). While the approaches to discourse analysis differ, they all study language and meaning. Boos (2005) claimed that discourse analysis in nursing research tends to be less scientific and methodologically consistent because the authors often describe the theory and analysis without focusing on descriptions of how they use discourse analysis theories or how the analysis has been concretely implemented. This article attempts to improve the balance of this analysis by devoting considerable space to understanding the structures and developing knowledge about how health providers collaborate with service designers.

This study thus aims to answer the following research question: Which discourses appear in the analysis of the employees' experiences of implementing a service design approach to change the processes used in a nursing home?

Focus group interviews were chosen as the research method to obtain knowledge about the employees`experiences using service design in a nursing home. This method is suitable for retrieving ideas and feelings related to experiences (Krueger & Casey, 2010). Focus group interviews is also suitable for establishing insights into the underlying factors and conditions related to people's opinions and experiences (Eines & Thylen, 2012) through various discourses held during the process.

#### 2.2.1 Participants and data collection

All nurses (*N *=* *13), nurse assistants (*N *=* *36), and cleaning ladies (*N *=* *3), who had been working at the nursing home for at least 1 year, were invited to participate in the study. The participants were given written information about the aim of the study and recruited by managers at the nursing home. Eleven employees agreed to participate in two different focus group interviews held at the midway point of the test period in 2015.

The same group of employees (*N *=* *52) were also asked to participate after the test period. However, only six employees attended the last focus group interview, which was conducted about 1 month after the test period. The informants were divided into three heterogeneous focus groups each containing five to six employees (Table 1). Members of each group were recruited from different departments; thus, each group comprised a mixture of nurses and nurse assistants.

**Table 1:** The study participants

Description of the population (*N* = 52)	Recruited informants midway in the test period	Recruited informants after the test period
13 nurses 36 nurse assistants 3 cleaners	First focus group interview: Three nurses (females) Three nurse assistants (females) Second focus group interview: Two nurses (females) Three nurse assistants (females)	Last focus group interview: Two nurses (females) Four nurse assistants (Three females and one man)

The interviews were conducted by the main author and a research assistant at the nursing home and lasted for 55–70 min. The main author recorded and transcribed each focus group interview. An interview guide was used to maintain a focus on the experiences of participating in a service design process and on the participants' involvement in the project.

The focus group interviews were conducted by two researchers. Before commencing the focus groups, the Norwegian Social Science Data Services was informed. The transcriptions provided considerable material related to the employees' experiences of using service design. The material was then analysed with a discourse‐oriented view based on their vocabulary and colloquialisms to determine how employees formed their understandings of service design process.

#### 2.2.2 Data analysis

Discourse analysis is a growing field in qualitative research, which is historically related to linguistics but also combined with critical theory, sociology, and psychology (Buus, 2005). Potter (2004) described discourse analysis as a methodological approach focusing on the relationships between what is expressed verbally, bodily, or textually, and other social elements in the context where the expression is found. In this article, discourses are understood as ways employees in nursing homes describe their experiences verbally using service design, which includes understanding the concepts, values, interests, and power constellations belonging to this context. The findings were analysed using discourse analysis with a sociological psychological anchoring (Potter, 2004; Potter & Hepburn, 2005). Discourse analysis can only create representations of reality and can never mirror the real experiences by the employees in this project (Jørgensen & Phillips, 1999).

The transcriptions of the three different focus group interviews were read through several times to establish an overall impression of what the employees conveyed. These aspects were highlighted with a marker in the text, shown as “comments”. Guided by the interview guide, analytical units and discourse themes were then identified, while retaining cohesion in the text and in the social structure to which they belonged (Buus, 2005) Table 2.

**Table 2:** Structures of the interview guide

Themes	Questions
Experiences working with the project	Please share your experiences of the new way of working at the nursing home
Collaboration between healthcare providers and service designers	Please talk about your experiences of collaborating with the service designers within the project

The thematic explanation of the material shown in Table 3 provides a basis for identifying discursive configurations of how the employees experienced the service design. Themes that contributed to these discourses were identified on individual and group levels and across various professional affiliations. The analysis resulted in the four discourses themes shown in Table 3. However, these discourses conflict because they indicate both positive and negative experiences.

**Table 3:** From text to themes

Text	Analytical units	Notes about how the quotes were expressed and analysed	Discourse themes
It was so interesting to think in a new way using creative methods and lots of paper and post‐its. It was a lot of fun!	Enjoyable working together using inspiring methods. Service designers use creative methods.	Different informants talked enthusiastically about how to collaborate with other professionals using new creative methods.	Discourse about the employees' engagement increased when using creative methods to focus on solutions
I know what to do now. I also think we understand each other's responsibilities at work in a better way now.	Better described roles and responsibilities. Better understanding among the staff.	Nurse assistants and nurses talked about how better‐ defined roles helped them understand each other's responsibilities at work in a better way.	Discourse about the recognition of competences.
I and several of my colleagues have talked about how these people, who never have worked with health care, can give us good advice.	Scepticism related to the service designers' lack of knowledge of health services.	Nurses and nurse assistants showed their scepticism related to the service designers' lack of knowledge of nursing home services both verbally and bodily. They did not like the way outsiders could develop effective interventions in the nursing home.	Discourse about scepticism—distancing themselves from the project
Basic information is necessary. Then we could have been better prepared all the time, and problems could have been resolved earlier.	Need for more information. How staff feel prepared to address problems during the process.	Some informants expressed that a negative feeling of not being informed and involved in the project was related to weak management of the project.	Discourse about not feeling informed and involved.

## 3 FINDINGS

The following sections present four discourses themes in detail.

### 3.1 Discourse about the employees' engagement increased when using creative methods to focus on solutions

The employees said they were initially very enthusiastic and positive about the project, because the service designers spent considerable time talking to them, which heightened their feeling of involvement in the project. The collaboration with service designers was described as a new approach which used various creative methods. The employees had positive perceptions towards this new approach because it was unlike methods that had previously been implemented at the nursing home. When asked about their experiences attending the workshops or other activities organized by the service designers, one nurse assistant responded as follows:

It was so interesting to think in a new way using creative methods and lots of paper and post‐its. It was a lot of fun!

Even though nurses and nurse assistants said they were not used to visualizing challenges and solutions with markers, notes, pictures, and images, they stated that “it was easier to see solutions than problems” because the service designers focused on solutions. Several of them also highlighted improved collaboration across the various departments at the nursing home and some pointed out the importance of using each other's expertise in a constructive manner.

During the workshops with colleagues and service designers, the employees expressed a desire to contribute to finding solutions and work as a group in a smarter way:

Nurse We worked very hard! It was a lot of fun to work in a creative way with the service designers.

Some nurse assistants described feeling positive because the project focused on increasing the quality of services rather than focusing on what was not working well.

Nevertheless, the employees agreed that service designers had “awakened them” and increased their awareness about how and why existing procedures and routines should be changed. Several mentioned how the new “well‐being team” helped to prioritize activities for the patients. The nurse assistants stated that the patients' needs had been a focus earlier, but the whole group of employees were now feeling more responsibility and reflected towards the patients, which reflected developed this thread as an important theme:

A nurse assistant We really increased the focus on how important it is for the patients' lives to feel well at the nursing home.

The employees often used the phrase “we” when emphasizing the value of some expressions and, overall, the analysis of the findings showed that the employees became more motivated and engaged when they collaborated using creative methods and focused on solutions instead of ongoing challenges. The participants used words such as “fun” and “creative methods,” which reflected a feeling of having contributed something new themselves. This language helps to create the experience and understanding of what the collaboration with service designers was and could be. Here, the experiences created something positive that was constructed as a social experience within a group, which could only be identified with a discourse‐oriented focus.

### 3.2 Discourse about the recognition of competences

When asked about why they liked the focus of the new model to emphasize the individual employee's resources, most of the employees said they liked the way the service designers focused on using the employee's resources and expertise, which had previously been lacking:

Nurse I think you have more to offer as a nurse if you feel competent. It feels great when someone asks what you would like to work with.

Through their language, the nurses and nurse assistants created an understanding that the innovation contributed to defining their competences, skills, and tasks, which improved their confidence in their work, something that benefits both the employees and the patients. Using their language to communicate also enable the employees to succeed in creating and supporting an experience of a social context. The participants mentioned that the new model more clearly defined their duties and responsibilities and that they felt important and more competent:

Nurse assistant We take more responsibility during the day, prioritising the tasks in a better way.

Nurse We know what to do and which role and responsibility each of us has.

Nurse I've got time to do what I have to do. I know better my responsibility as a nurse. It's a good feeling.

Nurse assistants said they had a better understanding of what the nurses actually needed to do, knowledge which was not quite clear previously:

Nurse assistant The nurses have become more visible and it's easier to understand their responsibilities.

Nurse assistant It is better both for them and us. I think we've got a better understanding of the different professions.

Notably, the participants did not use “they” and “them” when discussing the positive aspects of their better defined roles, responsibilities and tasks and they used “we” and “us” to emphasize the importance this had for the employees. This way of speaking creates and supports a notion of community and inclusiveness, where each professional's special competence forms a necessary piece in the nursing context of this project. The nurses and nurse assistants both highlighted the improved understanding between the professions that the new model brought. These findings indicate the importance of focusing on the employees' individual competence and resources to develop engagement and enthusiasm in a nursing home.

### 3.3 Discourse about scepticism—Distancing themselves from the project

The service designers and their methods did not inspire all employees. While some found it exciting that an outside team of service designers wanted to help them develop better health services, others focused more on the challenges that arose when they were required to collaborate with the service designers:

Nurse assistant We have talked about how these people, who never have worked with healthcare services, are able to give us good advice.

The nurse assistant distanced herself from the service designers by referring to them as “those people”. The use of “these people” also highlighted their scepticism towards the service designers. Several employees mentioned that such attitudes about working in a project were often “contagious”; some tried to make the whole group revolve around thinking positively, while others spread more negative attitudes:

A nurse Some are always negative and in a bad way, they are recruiting more who think like them. Then it is almost impossible to achieve. Conversely, if you have one or two with lots of guts and positivity, it's amazing what you can make out of it!

One nurse assistant mentioned a phase of the project that led to much opposition in the group of employees:

The first few weeks when we tried out the new model was a chaos!

This chaos caused frustrations among the employees and reduced their enjoyment participating in the project. However, after making some adjustments together, which contributed to a better situation, the chaos dissipated. The employees discussed the importance of having the management understand their frustrations. However, several of the participants said they were demotivated by the lack of continuous evaluation and adjustment during the process. These findings showed the necessity of understanding how to motivate each employee during an innovation process.

### 3.4 Discourse about not feeling informed and involved

Most of the employees with various professional affiliations said that having the management more involved in the process, so they could provide employees with more information, would have strengthened their commitment to the project. Several employees discussed how information and involvement was needed to maintain their motivation and enthusiasm for the project over time:

Nurse assistant The project has not ended, but we are somehow not “inside” and know almost nothing about how to implement the good ideas.

Nurse Basic information is necessary. Then we could have been better prepared all the time and problems could have been resolved earlier.

The descriptions showed that the employees' involvement decreased following their involvement in the initial phases of brainstorming, the workshops, and the concept development to the test period when they tested the new model at the nursing home. Some of the employees tried to give oral and written feedback to their managers without any effect. One nurse assistant described her frustration:

You write many messages in a book, but they seem to have no impact. Nothing happened so I gave up.

The nurses and nurse assistants both missed receiving information and communicating with their managers, and explained that the management's involvement and presence reduced when the new model was being tested. One nurse said the following about the importance of the manager's role in the project:

Managers who answer are very important, even though they do not know everything.

The employees pointed out that there was no correlation between their expectations in the initial phase and how the managers run the test period. The participants' descriptions showed a need for continual adaptations of the model in the period of testing the new model. Several employees also described their scepticism towards the project because they felt the methods were led by service designers who had little knowledge of a nursing home's function and organization. Most of the employees also explained that their enthusiasm decreased following the testing period because the new model combined fewer creative and inspiring activities than before:

Nurse assistant It is such a shame that we no longer work in groups with notes and drawings.

Many employees also felt that they lacked sufficient knowledge about service design, which may have affected their involvement in the process. These findings show the need to ensure strong management involvement.

## 4 DISCUSSION

Innovation processes are complex, requiring the involvement of several factors to succeed (Johnsen & Paalshaugen, 2011). This study examined discourses among employees, which could promote or curb the innovation processes. The discourses need to be understood in light of the context to which they belong. Working interprofessionally in a nursing home could be considered complex because of the various values, interests, power constellations, and social structures that influence the exchange of experience. Discourses must therefore be understood as functions, structures, and variations in the reality described by the participants (Potter, 2004).

The service designers' creative and visual methods generated enthusiasm among the employees in the first phase of the project. A creative approach contributed to positive experiences, prompting the participants to use adjectives such as “fun” and “amazing” when describing the methodology. The discourse describing the employees' increased engagement described the use of creative methods that focused on solutions, thus indicating that these methods can motivate employees to participate in challenging innovation processes (Stickdorn & Schneider, 2011). The enthusiasm related to the use of creative approaches was highlighted by employees of both professions involved in the study. The participants also appreciated the way the service designers gathered knowledge of their personal qualities and professional resources to incorporate in the design of the new model to improve existing services. Such employee involvement promotes the use of the employees' knowledge and skills in a constructive way (Brady & Cummings, 2010; Ramirez, West, & Costell, 2013; Willumsen & Ødegård, 2015). Using a service design approach thus aimed to improve a common social structure and culture in the nursing home. The employees` feeling of ownership of the new solutions worked out through creative methods, also increased the degree of inter professionally collaboration in the different departments.

Previous research has highlighted the challenges related to understanding other's roles and responsibilities among different professions (Jacobsen, 2010; Lund, 2003; Willumsen & Ødegård, 2015). The discourse of recognition in this study indicates that closer collaboration across the professions strengthened the participants' professional identities by providing more clearly defined roles, responsibilities, and tasks. Although not all of the participants used “we” as a pronoun instead of “them,” several pointed out that the different professions at the nursing home had developed a clearer understanding of each other's roles and responsibilities. The need to fight for professional autonomy seemed to decrease because the new model helped the group of employees demonstrate the need for differentiated tasks and responsibilities across the professions. The discourse of recognition showed that the employees' involvement in the project increased because the service designers focused on each employee's skills. However, the way the nurses and nurse assistants mentioned “those people” when referring to the service designers showed lack of respect for the service designers' competence in the healthcare field. Because the healthcare providers are not used to collaborating with professions without any healthcare competence, this could explain their misunderstanding of the need of the service designers' competence. To innovate health services interprofessional competence is needed to develop more creative solutions (Willumsen & Ødegård, 2015). This study show how competence and interprofessional collaboration can not only be observed but it can also occurs verbally, like a discourse.

The discourses showed some contradictions in the innovation process, where commitment and motivation both promoted and hampered the progress. The employees were motivated and worked hard when they were involved in the first phase of the project. However, too little involvement and information from their manager generated some scepticism among the employees, which distanced them from the project. The involvement of the employees should be maintained through ongoing workshops and open communication during the entire project. This finding supports that of previous research (Kramer et al., 2007; Robinson, Williams, Dickinson, Freeman, & Rumbold, 2012; Smith, Hampson, Scott, & Bower, 2011; Thakur et al., 2012), which pointed out the importance of having managers who support and communicate with their employees during an innovation process. The discourses highlighting positive experiences related mostly to the project's first phase, where the staff worked closely together with the managers and service designers to develop an effective organizational model.

The discourses highlighted challenges in the innovation process as a result of inadequate information and involvement after the initial phase of the project, which affected the employees negatively. Informants often used “we” and “them” when describing experiences related to collaboration with their managers: “we” was typically used when describing positive experiences, for example, when the managers “understood us” and “them” was used when the participants described the frustrations associated with misunderstanding in a chaotic phase of the project. Hendy and Barlow (2012) highlighted the importance of communicating with employees about why changes are necessary, which perhaps explains why the employees' engagement was renewed after receiving information from their managers about how they could adjust and develop the new model further. Communication, involvement, and continuous evaluation are important qualities in service design and in leading innovation processes (Battilana, Gilmartin, Sengul, Pache, & Alexander, 2010; Brady & Cummings, 2010; Stickdorn & Schneider, 2011; Vogus & Welbourne, 2003).

The discourse about too little information and involvement also draws attention to the challenges in the transition between the development of a model and its implementation in the organization in daily operations (Battilana et al., 2010; Bessant, 2005). The project highlighted the need for closer collaboration between service designers, managers, and employees in all phases of a project. The service designers were hired to develop a new model with the patients, relatives, employees, and management of the nursing home.

The findings of this study also show that reduced involvement led to doubts about the service designers' lack of expertise about healthcare services. Involving all stakeholders in the entire process would thus reduce confusion, doubt, and misunderstanding among those participating in the process. Thakur et al. (2012) described the complexity of implementing innovative solutions in practice and the findings of this study suggest that service design in healthcare should focus on both the early and later phases of the innovation process to successfully implement new and improved solutions.

Innovations include developing interventions to overcome the challenges (Andreassen, 2011). The strategy of using service design in this project seems to have succeeded, with the focus on a better use of resources and the involvement of employees in the early phases of the project. The participants described their participation in the development of the new concept of organization and the roles and responsibilities of employees with the service designers as positive. Innovation comprises learning and new understanding through testing and learning from mistakes (Bessant, 2005), which was echoed by several participants, as shown in the following example:

Nurse The model could be great, but we need some more adjustments.

This learning is also a result of the discourse of recognition in line with the service designers' philosophy about continuously evaluating, adjusting, and testing new solutions continuously (Stickdorn & Schneider, 2011). If a solution does not fit, it should be modified before being retested. Discourses about the increased engagement using creative methods to focus on solutions and the recognition of competence show how cocreation and involvement are essential for maintaining enthusiasm among the employees in an innovation process. The findings also show that employees are motivated in different ways. Discourses are structures and variations (Potter, 2004), where the variations in discourses in this study show how employees get motivated differently during the project.

The findings of this study also show the importance of integrating different perspectives to develop better services that address the patient's needs. Health professionals often work paternalistically to create high‐quality services; however, user involvement and insight about patients' real needs are increasingly becoming central to the development of healthcare services (Ministry of Health and Social Services in Norway, 2008, 2011, 2012).

### 4.1 Strengths and limitations

One limitation of this study is that it does not focus on the success of the service design, but rather on the employees' discourses about it. Discourse analysis indicates the effect of positive or negative attitudes. Thus, if one of the participants of a group described something as positive, the other members of that group tended to agree. In addition, these discourse analyses also showed how negative experiences often are placed within the professional group or at an institutional level, through statements such as “we believe” or “this applies to us”.

However, the discourse‐oriented approach has underscored the crucial role of language in constructing the health providers' experiences and explanations and the importance of the verbal process of innovation and development work. Meanings and knowledge about healthcare, today and in the future, are largely constructed through language. These constructions form the reality where decisions and practices happen. Therefore, discourse‐oriented research and discursive competence are crucial in healthcare development. Nevertheless, we consider that a combination of discourse analysis and observation would have strengthened the credibility of the findings, especially since the focus group interviews included only a handful of employees. More participants, especially in the focus group interview conducted after the test period, could have provided a greater variation in the findings. Too few participants in the study could also lead to representation of especially critical or satisfied participants in the focus groups.

The findings show how a service design process creates energy and develops new ways of thinking. The approach of this study focuses on how the participants articulated their experiences, rather than focusing on real truths and individuals' lived experiences of the services (Buus, 2005). A focus on language, views about professional knowledge and service development are highlighted in a contextual perspective of reality and knowledge. The professional skills often become difficult to point out, problematize, and develop because of the tacit knowledge among the health professions. Discourse‐oriented studies of employees' experience using service design is intended as a contribution to innovation processes, thus strengthening the reflection among the employees and management in healthcare services.

Representations of different realities were expressed when the participants disagreed. While discourse analysis does not try to uncover “realities,” it may be worth identifying statements that highlight the different experiences by the participants in this project. It might also be worth finding out why some of the statements were more accepted and dominant than others within a group or across the participants; for example, both nurses and nurse assistants pointed out that the project created a better understanding across the professions because their roles and responsibilities were clearly defined.

## 5 CONCLUSION

The aim of this study was to explore nurses and nurse assistants' experiences using service design in a nursing home. The discourses in this study show that no single actor or factor determines the degree of success in an innovation project in a nursing home, where the employees face continual challenges. The collaboration with service designers and implementing new and creative methods was initially found to be a positive experience among the employees, which increased their enthusiasm and commitment among employees developing and testing a new service model for better practice. The focus the service designers placed on each employee's resources seemed to help the nursing home develop a common structure for inter professional collaboration understanding and to develop a new level of respect for each other's competences.

However, the collaboration and the quality of communication between the management and employees failed to maintain enthusiasm and commitment throughout all phases of innovation processes. Discourses about positive experiences and challenges in this project thus highlighted the importance of continuous collaboration as well as the involvement of patients, employees, service designers, and managers in all phases from planning to implementing a new model. Future research should focus on how service design approaches could be used to implement new solution in healthcare services.

## AUTHOR CONTRIBUTIONS

All authors have agreed on the final version and meet all of the following criteria (recommended by ICMJE http://www.icmje.org/recommendations/):

- Substantial contributions to the conception or design of the work; or the acquisition, analysis, or interpretation of data for the work
- Drafting the work or revising it critically for important intellectual content
- Final approval of the version to be published
- Agreement to be accountable for all aspects of the work in ensuring that questions related to the accuracy or integrity of any part of the work are appropriately investigated and resolved
